# Coexistent sarcoidosis and lymphangioleiomyomatosis in a patient with cystic lung disease

**DOI:** 10.1002/rcr2.389

**Published:** 2018-11-28

**Authors:** Sarah Cullivan, Peter De La Harpe Golden, Deirdre Doyle, Kishore Kumar Doddakula, Louise Burke, Desmond Michael Murphy

**Affiliations:** ^1^ Department of Respiratory Medicine Cork University Hospital Ireland; ^2^ Department of Pathology Cork University Hospital Ireland; ^3^ Department of Radiology Cork University Hospital Ireland; ^4^ Department of Cardiothoracic Surgery Cork University Hospital Ireland

**Keywords:** Cystic lung disease, lymphangioleiomyomatosis, sarcoidosis

## Abstract

A 45‐year‐old lady presented acutely with pleuritic chest pain, haemoptysis, and dyspnoea. Her background was significant for a 1.4 cm renal angiomyolipoma, and she was an ex‐smoker without any relevant family history. A computed tomography (CT) pulmonary angiogram was negative for a pulmonary embolism but demonstrated diffuse cystic change throughout both lungs. A bronchoscopy confirmed a normal endobronchial tree, and pulmonary function tests demonstrated moderate airways obstruction, with reversibility and a normal diffusion capacity for carbon monoxide (DLCO). A video‐assisted thoracoscopic surgery (VATS) lung biopsy showed non‐caseating granulomas, and serum angiotensin converting enzyme (ACE) was elevated consistent with a diagnosis of pulmonary sarcoidosis. Further sectioning indicated focal areas that stained positive for Human Melanoma Black 45 (HMB‐45), confirming lymphangioleiomyomatosis (LAM). A diagnosis of cystic lung disease secondary to coexistent sarcoidosis and LAM was made.

## Introduction

This is an interesting case of coexistent lymphangioleiomyomatosis (LAM) and pulmonary sarcoidosis in a 45‐year‐old lady. This case highlights important clinical, radiological, and physiological features of both conditions and suggests a potential shared disease mechanism.

## Case Report

**Figure 1:**
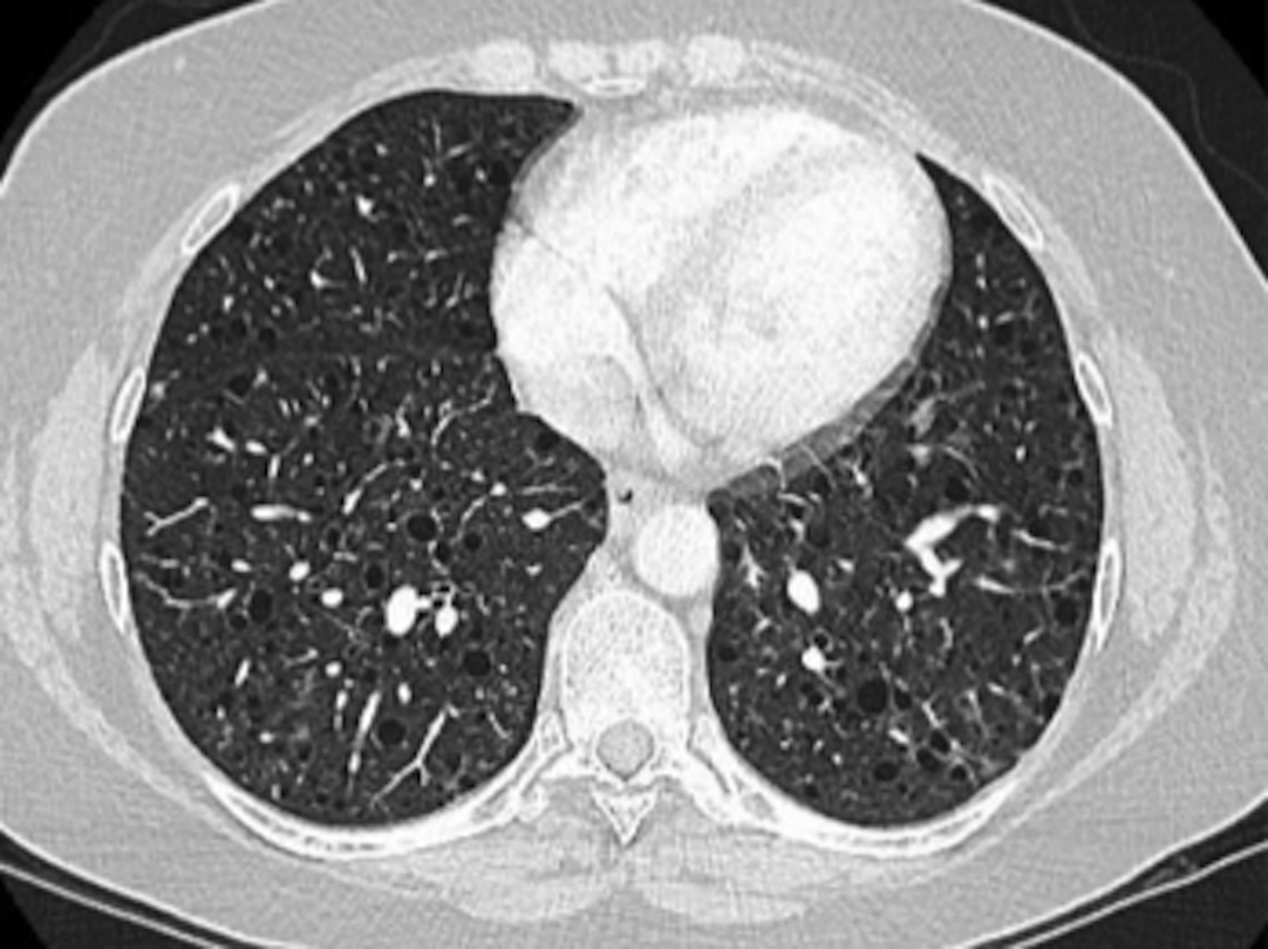
A 45‐year‐old lady presents with acute dyspnoea. Computed tomography (CT) pulmonary angiogram demonstrates multiple bilateral cysts that are evenly distributed throughout the pulmonary parenchyma. Ground‐glass nodules were noted; there were no solid parenchymal nodules or lymphadenopathy.

A 45‐year‐old lady was admitted with acute pleuritic chest pain, haemoptysis, and dyspnoea. Her background was significant for a 1.4 cm left renal angiomyolipoma, myofascial pain syndrome, and depression. Regular medications included a combination umeclidinium and vilanterol inhaler and escitalopram. She was an ex‐smoker, with a 5 pack‐year history, and denied any relevant family history or occupational exposures. A computed tomography (CT) pulmonary angiogram was performed on admission. This was negative for a pulmonary embolism but demonstrated diffuse, well‐circumscribed cystic change throughout both lungs, with no zonal predominance. Small foci of ground‐glass change were noted to be interspersed between the cysts. There were no associated parenchymal nodules or lymphadenopathy (Fig. 1). She was treated for a lower respiratory tract infection and subsequently referred to a tertiary centre for further assessment.

**Figure 2:**
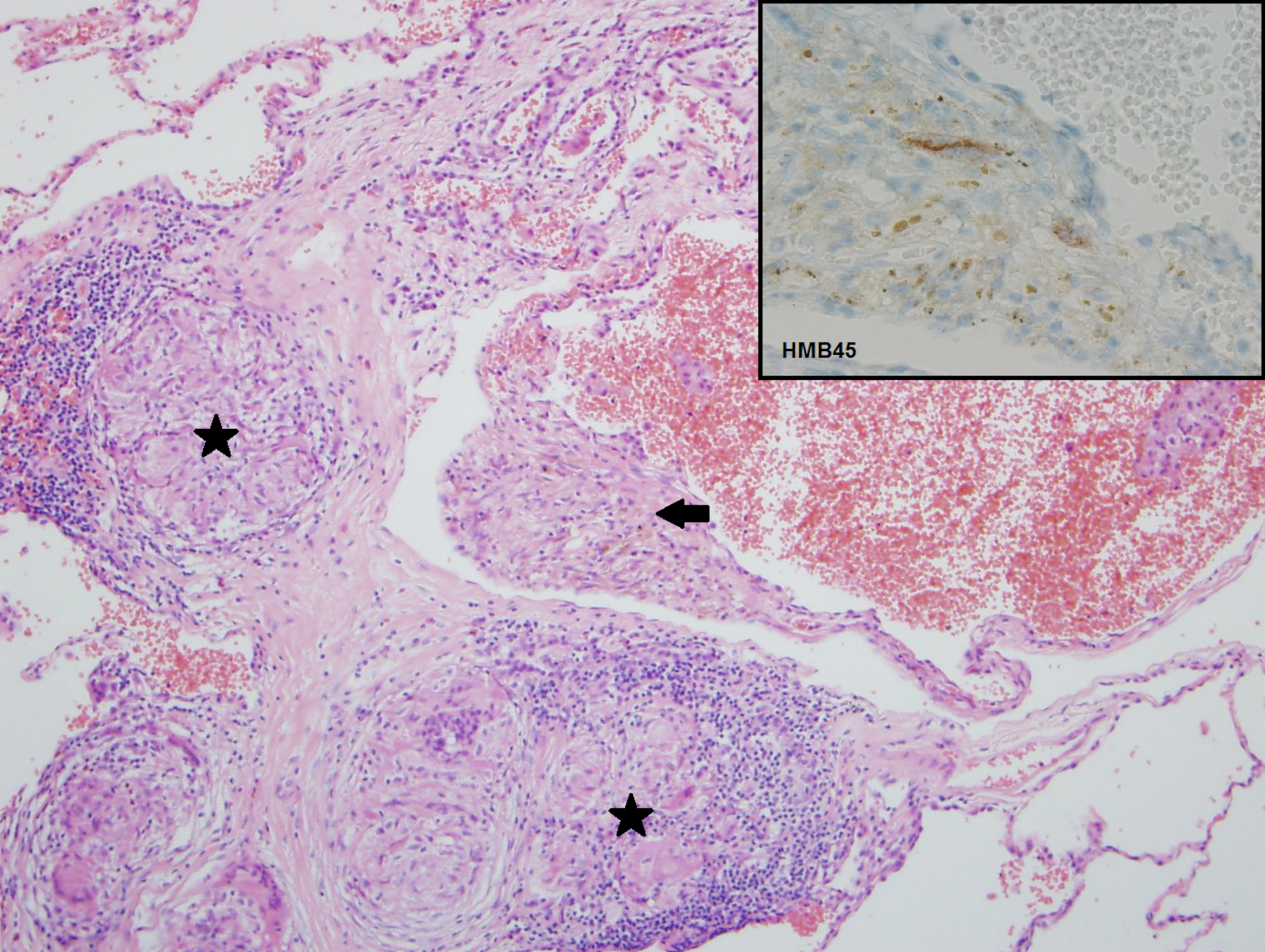
A 45‐year‐old lady presents with acute dyspnoea. Right upper lobe biopsy demonstrates multiple thin‐walled cysts. Areas of smooth muscle proliferation that stain focally positive for HMB‐45 are noted (arrow), which is diagnostic of lymphangioleiomyomatosis. Superimposed non‐necrotizing granulomatous inflammation (stars) is present in a lymphatic distribution, consistent with pulmonary sarcoidosis.

On review, she reported modified medical research council (mMRC) grade 2 dyspnoea at baseline. A bronchoscopy demonstrated a normal tracheobronchial tree, and a bronchoalevolar lavage was auramine stain and tuberculosis culture negative. Autoimmune serology was also unremarkable. Pulmonary function tests demonstrated forced expiratory volume in 1 second (FEV_1_) of 1.79 L (62%), forced vital capacity (FVC) of 2.33 L (70%), a positive bronchodilator response of 390 mL (23%), and a normal DLCO. A diagnosis of tuberous sclerosis‐associated LAM was subsequently suspected based on a history of a renal angiomyolipoma and the presence of cortical tubers on a screening magnetic resonance imaging (MRI) brain, and a lung biopsy was requested for confirmation. This initially demonstrated predominantly non‐caseating granulomas. She was also subsequently found to have an elevated serum ACE of 68 U/L (reference range 0–45 U/L). A diagnosis of pulmonary sarcoidosis was made. Further biopsy sectioning demonstrated focal areas of positive HMB‐45 staining, confirming LAM (Fig. 2). A diagnosis of cystic lung disease secondary to coexistent sarcoidosis and LAM was established.

Our patient was switched to an inhaled corticosteroid and long‐acting beta agonist given reversibility on spirometric testing and is monitored closely in the respiratory outpatient department. At present, she is not prescribed targeted therapy for sarcoidosis or LAM and remains clinically stable from a respiratory perspective.

## Discussion

This case describes an incidental finding of coexistent LAM and sarcoidosis in a female patient. LAM is an orphan lung disease that classically effects females of child‐bearing age and may result in progressive cystic lung disease and respiratory failure. It can occur sporadically or in association with tuberous sclerosis complex (TSC), and both are characterized by mammalian target of rapamycin (mTOR) dysregulation 1. Alternatively, the incidence of pulmonary sarcoidosis is highly variable and is associated with a diverse clinical course and prognosis 2. The priority in this case was to ascertain if abnormal clinical, physiological, and radiological parameters were secondary to LAM, sarcoidosis, cigarette smoking, asthma, or a combination of all four as this will have important implications for future therapeutic choices if her respiratory disease progresses.

Imaging in LAM typically demonstrates multiple, thin‐walled cysts with no significant zonal predominance as described in this case 1. Cystic lung disease can also occur in sarcoidosis; however, this is not common and usually occurs in the upper and hilar zones in the context of advanced fibrosis 2. Therefore, cystic lung disease in this case is presumed primarily due to LAM given classical radiological features. Regarding treatment options, sirolimus, an mTOR inhibitor, is recommended for patients with LAM and an FEV_1_ less than 70% predicted or declining lung function 3. Obstructive spirometry in this case is potentially confounded by a prior smoking history or coexistent asthma. Therefore, we elected to commence inhaler therapy, monitor regularly, and subsequently commence sirolimus if there is any subsequent deterioration. Interestingly, there are cases of LAM and sarcoidosis overlap in the literature and emerging data to suggest a shared pathological mechanism between the two conditions 4, 5. It is hypothesized that dysregulation of the mTOR pathway could also play an important role in the pathogenesis of sarcoidosis 6. Early data suggest that dysregulated mTOR signalling is implicated in macrophage granuloma formation and sarcoidosis progression 7. Currently, there is inadequate available data to recommend mTOR inhibitor therapy in other respiratory conditions, and additional research is required to clarify the role of these agents.

Finally, this case reinforces the persistent utility of lung biopsy and pathology in cases of cystic lung disease. While serological markers such as serum vascular endothelial growth factor D (VEGF‐D) are highly specific and sensitive for LAM when available, if there is any clinical doubt, then lung biopsy should be considered 3. Importantly, the diagnosis of sarcoidosis was made post‐lung biopsy and was not suspected prior to this.

This is an interesting case of coexistent LAM and sarcoidosis and complements existing data that suggest a link between mTOR dysregulation and pulmonary sarcoidosis.

## Disclosure Statement

Appropriate written informed consent was obtained for publication of this case report and accompanying images.
